# Forms of sexual violence perpetrated in conflict and post-conflict settings against South Sudanese men resettled in two communities in Uganda: an exploratory qualitative study

**DOI:** 10.1186/s13031-023-00544-7

**Published:** 2023-10-18

**Authors:** Tosin Olaluwoye, Elizabeth Hoban, Joanne Williams

**Affiliations:** 1 Deakin University, Geelong, Australia; 2 Swinburne University, Melbourne, Australia

**Keywords:** Sexual violence, Male survivors, Conflict-related sexual violence, Male rape, Refugees, South Sudan

## Abstract

**Background:**

Consideration for men as survivors of sexual violence in conflict and post-conflict settings has gained some prominence in the last decade. There remains a paucity of empirical data on forms of sexual violence from the survivors’ perspective, and no study has considered the context of the 2013 South Sudan conflict specifically.

**Methods:**

This paper reports the findings of an exploratory qualitative study on the forms of sexual violence perpetrated against men in conflict and post-conflict settings, with the survivors as the main participants. A purposive sampling technique was used to recruit 26 South Sudanese male sexual violence survivors who have resettled in two refugee resettlement communities in Uganda since the onset of the 2013 South Sudan conflict. In-depth semi-structured interviews were used to collect the data from the male survivors. Six humanitarian aid workers who support sexual violence survivors also participated as key informants. Thematic data analysis was performed on the qualitative data.

**Results:**

The 26 survivors reported experiencing eight direct and two indirect forms of sexual violence in conflict and post-conflict settings. The direct forms include (1) striping men naked; (2) male rape; (3) exchange of sex for favours; (4) forcing men to rape other people; (5) genital mutilation; (6) genital beating; (7) insertion of objects into men’s anus, and (8) taking men as wives. The indirect forms were forcing men to witness the rape of a female relative and forcing men to cheer or assist during the rape of other people.

**Conclusion:**

To maximize positive health outcomes for survivors, stakeholders must consider both direct and indirect forms of male-directed conflict-related sexual violence in policy and practice.

## Introduction

Sexual violence is one of the many negative events that characterised armed conflicts in ancient and modern history [1, 2]. Krug and colleagues defined sexual violence as “*any sexual act, attempts to obtain a sexual act, or acts to traffic for sexual purposes, directed against a person using coercion, harassment or advances made by any person regardless of their relationship to the victim, in any setting, including but not limited to home and work”* [3]. To be regarded as conflict-related sexual violence (CRSV), a sexual act must have at least two of the following links to conflict: temporal, geographical or causal links [4, 5]. CRSV has gained prominence on the international agenda since the 1994 Rwandan genocide and the 1992–1995 ethnic cleansing in Yugoslavia [6]. Although there is evidence that women and children are disproportionately more affected by CRSV, there is a growing body of literature on sexual violence perpetrated against men in conflict and post-conflict settings [7–12]. Sexual violence against men has been reported in at least 25 armed conflicts globally [13].

There is no consensus in the literature on what constitutes CRSV (including against men). However, some of the forms of CRSV that have been reported include anal or oral rape, enforced incest, forcing men to rape other men, enforced sexual intercourse with dead bodies and forced masturbation [8, 14]. Other examples are castration and enforced sterility, forced nudity, intentional trauma to genitals, being forced to traumatise other victims’ genitals, genital mutilation, exchange of sex for a favour, and sexual comforting [11, 13, 15]. Some scholars have discussed the concepts of direct and indirect victimhood. Direct victims are those who experienced the act, while indirect victims share a relationship with direct victims and suffer because of the harm perpetrated against the direct victims. Synonyms used to describe indirect victims of CRSV include “connected” or “secondary” victims [10, 16, 17].

The acts of male-directed CRSV reported above, were based on extrapolations from data obtained in studies with predominantly female survivors [11, 18] and information provided by humanitarian agencies’ staff [11, 14]. Chynoweth and Schulz conducted focus group discussions with male survivors of CRSV to collect data on the forms of male-directed CRSV (including survivors already receiving support for their experiences) [8, 10]. However, to the authors’ knowledge, no study has conducted in-depth interviews with male CRSV survivors who had not previously received support.

The South Sudan armed conflict that started in 2013 was regarded as the largest refugee crisis in Africa and the third largest in the world, after Syria and Afghanistan [19] and was characterised by human rights abuses, including sexual violence [20]. The conflict led to the displacement of over five million South Sudanese , 46% were men, either to the United Nations Protection of Civilian sites within South Sudan or to neighbouring countries like Uganda, Sudan, Kenya, Ethiopia, Congo, and the Central African Republic [21]. Despite the magnitude of the South Sudan armed conflict, there is scant documentation of CRSV perpetrated against men within South Sudan, during their flight from their country and in post-conflict refugee resettlement communities in neighbouring countries.

This paper provides evidence about the forms of CRSV perpetrated against South Sudanese men in conflict and post-conflict (resettlement) settings during the South Sudan conflict that lasted between December 2013 and February 2020. The data presented is the perspective of survivors’ who had not previously received support for their experiences.

## Methods

The study was conducted between January and April 2019 in Imvepi and Rhino resettlement communities in the Arua district, West Nile, Uganda. At the time of the study, Uganda provided refuge for 42.6% of South Sudanese refugees that fled their country since the onset of the 2013 conflict [21]. The relatively large number of South Sundanese refugees in Uganda informed the choice of the study location. Twenty-six South Sudanese male sexual violence survivors who had relocated to Rhino and Imvepi communities in Uganda since the 2013 South Sudan conflict started participated in the study. Although the South Sudanese population have experienced previous wars, this study focused on recent events so that participants could recall and describe their experience in as much detail as possible. Six humanitarian aid workers who provided support for survivors of sexual violence were also interviewed. A male research assistant (RA) was employed to support the primary researcher (first author) during community entry, help translate the consent form to local languages and act as an interpreter during interviews with participants who preferred to speak languages other than English. The RA had lived in the Arua district for over five years, understood the local context well, and could communicate in English, Arabic, Swahili, and other major South Sudanese languages. The RA was trained by the primary researcher on the subject, participant recruitment, data collection strategies and the importance of privacy and confidentiality.

The research team moved into the Arua district four weeks before participant recruitment commenced to develop trust relationships with community/youth leaders and other stakeholders and learn about the South Sudanese culture, beliefs, norms, and interests [22]. The community and youth leaders acted as gatekeepers, helping to access CRSV survivors in a manner that protected the participants’ identity from the leaders, whilst ensuring the identity of survivors that declined participation. Once a person indicated their interest in participating in the research, the RA discussed the details of the study using the plain language statement and gave them another week to further consider participation. All but one male survivor who contacted the research team returned after one week. Participants were recruited using the snowball sampling technique. They chose pseudonyms (to ensure privacy and confidentiality) and gave verbal informed consent to proceed with the data collection.

In-depth semi-structured interviews were conducted with the participants and were audio-recorded and transcribed within 48 hours of collection. The interview guide included questions about their life in general since the onset of the conflict, their experience of CRSV, its effect on them and the type of support that sought for their experience. Participants were given their transcript to read to ensure respondent validation/member checking, and all transcripts were confirmed to be accurate. Inductive thematic analysis was conducted manually to extract relevant themes from the data [23]. The themes and subthemes that emerged from the analysis formed the headings and sub-headings of the results, presented below.

Ethical approvals were obtained from the Deakin University Human Research Ethics Committee (DUHREC) (2018 − 223); the Research Ethics Committee in the School of Social Sciences, Makerere University (REC 10.18.227); the Directorate for Disaster Preparedness and Refugees in the Office of the Prime Minister (OPM) in Uganda (OPM/R/41/1) and the Ugandan National Council for Science and Technology (UNCST) (SS 4829) (REDACTED FOR ANONYMITY). Other ethical issues considered include strategies to manage possible re-traumatisation, privacy and confidentiality, the use of verbal informed consent and compensation for participation.

## Results

The results of the thematic data analysis found that CRSV perpetrated against the male participants in conflict and post-conflict settings can be classified into direct and indirect forms (Table 1). Direct forms of CRSV included exposure of genitals, involvement of sexual intercourse, trauma to genitals and switching gender roles. Indirect forms of CRSV included being forced to witness sexual violence and being forced to show support for sexual violence.

**Table 1:** Forms of CRSV perpetrated against South Sudanese men who resettled in two resettlement communities since the onset of the 2013 South Sudan conflict

Themes	Subthemes
***Direct forms of CRSV***	
Exposure of genitals	• *Stripping a man naked*
Involvement of sexual intercourse	• *Male rape* • *Exchange of sex for favours* • *Forcing men to rape other people*
Trauma to the genitals	• *Genital mutilation* • *Genital beating* • *Insertion of objects into the anus*
Switching gender roles	• *Taking the men as wives*
***Indirect forms of CRSV***	
Forced witnessing of sexual violence	• *Forcing men to witness the rape of women in their lives*
Forced support for sexual violence	• *Forcing men to cheer/assist during rape*

### Exposure of genitals

#### Stripping men naked

Most participants reported that stripping men partially or completely naked was a common occurrence since the onset of the conflict. Partial stripping involved forcing men to remove all their clothes except underwear, while complete stripping entailed the removal of all pieces of clothing. In most cases, when perpetrators accosted their would-be victims, the first instruction they gave their captive (usually at gunpoint) was to strip off their clothes, as a way of subduing them. Smalling, a CRSV survivor, who was partially stripped in his house in South Sudan, captured this by saying:

*“Immediately they entered inside the house, what they did was to tell me to strip off my shirt and then they removed my shoes, they removed my trousers, I was just left with my pants”.*

In some cases, perpetrators considered stripping men naked was enough punishment for their victims and they did not inflict any other form of violence on the men. Such was the experience of Sane, who said:

*“They only removed our clothes… they did not say something like they want to rape us, but they left us like that”.*

Perpetrators who only stripped the men naked usually did so in the presence of other people, such as the survivors’ wives, children, and other family members, to humiliate the men. For instance, Luis, who was ordered to strip naked while his wife watched.

Regardless of the extent or intention of stripping, survivors felt ashamed and sexually violated by the act. According to Smalling (mentioned earlier):

*“The sexual violence I experienced there is like a human being who is matured, you need not strip somebody naked and move just naked and ladies moving just like that only putting on small clothes, and you are made to sit together with them…I just saw that as torture sexuall*y”.

While describing his experience, Smalling made repeated involuntary hand movements to cover his genitals each time he spoke about being stripped, even though he was fully clothed.

#### Involvement of sexual intercourse

##### Male rape

Male rape was one of the acts that sometimes followed the act of stripping men naked. Some survivors reported that they witnessed other men (including male family members) being raped, while others said they were raped. However, most of those who reported witnessing male rape, noted that they had witnessed it happen multiple times. Messi, who witnessed multiple male rapes in South Sudan and during flight to the refugee resettlement communities, gave this account:

*“Within Juba in South Sudan in 2016, so many men were raped that I saw. Their clothes were removed, and they were raped. Also, when we were moving towards Nimule because our plan was to go to the camp, we reached the middle, and those soldiers just stopped us on the way with guns. They said we should remove our clothes, and we had no option because if you don’t want to remove them, we would be killed. So, we removed our clothes, and they started raping people, including men.“.*

When asked about what they meant by rape, some participants noted that it involved the insertion of the perpetrators’ penis into the survivor’s anus. For example, Essien reported that:

“*The mistreatment they did sometimes is they remove their penis from their pants and put it in your anus when you are naked, but you cannot do anything… if they get you as a man who is against them in government, they play sex with you through your anus”.*

Luis said that he was stripped naked in the presence of his wife and then he was gang-raped twice by rebel fighters. Luis’s first experience was in South Sudan, and according to him,

*“They caught three of my friends and me and told us to remove our clothes and lay down. They were using guns, and we had to remove our clothes. Then, they raped the four of us. They raped us seriously and told us that we had to accept anything they were doing to us, or we would lose our lives”.*

His second experience was more horrifying, and it occurred in the bush and during flight to Uganda. Before a large group of rebel fighters raped Luis, they killed his two children and raped his wife, in his presence. According to Luis:

*“I met with a group of rebels in the bush* [during flight] *that came and surrounded me… those guys raped me also, and they were about 20 in number. So, I decided to surrender to them to take my life like that”.*

The humanitarian aid workers corroborated the male survivors’ rape stories and noted that male refugees were being raped in the resettlement communities. Doris, one of the humanitarian aid workers said:

*“Even when they get asylum here, abuse follows them. War come with such kind of things; you know. Women are raped, and men are raped within the resettlement. I have heard of rape cases that men reported since I started the operation”.*

### Exchange of sex for favours

The situation created by the conflict led to some South Sudanese men prostituting themselves to fighters, aid workers, fellow South Sudanese and Uganda nationals in exchange for favours like money, food, or freedom from captivity. While sex for release from captivity occurred in the conflict zones (including South Sudan and during flight to Uganda), the exchange of sex for money and food was more common in the resettlement communities. Rudiger, a male refugee, was asked if he had witnessed or experienced sexual violence in the resettlement community and he reported that he knew a man who offered sex as a means of survival.

*“One was a man who was not working, and people just come to him, pay him money, and sleep with him. The man is a refugee. The reason the man is doing so is because he is hungry. There is no food. The second thing is that there is no clothing he can wear. So that is why he is doing so to survive”.*

According to Mary, a humanitarian aid worker, men are not the sole perpetrators of sexual violence against men. In some instances, women who hold some power or have financial resources take advantage of their status and exploit poor men for their sexual satisfaction.

*“These are young men, and you will find the females are older than them. It is not like this is a real partner that this person has chosen, but this is an older person with some power or authority or something like they have money, and they will wish to force these young men. They are like taking advantage of them. We have some that have happened in the settlement. You find these women who have much money in South Sudan, and now they are in camp, and they will wish, you know, if you encourage someone to come and be with you in the family and tend to force sex. This has been reported to me”.*

#### Forcing men to rape other people

Another form of sexual violence noted by the South Sudanese men was being forced to rape other men or women. Participants reported instances of men being caught by rebel or government fighters during flight and instructed to choose another male captive to have anal sex with. In some situations, the men were forced to rape their mothers, daughters, or sisters while their captors watched. Neymar recounted two stories of enforced incest. In the first case, rebel fighters instructed Neymar’s friend to rape his own sister, and a refusal to commit the incest led to Neymar and his friend being raped themselves:

*“A friend of mine was caught by soldiers and he was told to remove his clothes. When he removed his clothes, they told him to rape his own sister. He ran towards me, asking me to please help him as he could not have sex with his own sister, but I told him I could not help as I was also in trouble myself. When those rebels saw that he ran to me, they decided to take the two of us, and they told the man, “since you do not want to rape your sister, we are going to rape the two of you”. From there, they raped my friend and me and left”.*

Neymar and his friend later joined a rebel militant group to take revenge on the soldiers who did this to them. Neymar’s second experience occurred in South Sudan when he witnessed a man who was forced to rape his mother. The man committed suicide after the incident as he could not live with the thought that he had committed incest against his mother.

Lacazette, a humanitarian caseworker, noted that his agency had supported a man who was forced to rape his daughter during the flight from South Sudan to Uganda and after the incident this man became suicidal.

### Trauma to the genitals

#### Genital mutilation

Although none of the participants had their genitals cut off, some reported that they had witnessed other men’s penis or scrotum being cut off by rebel fighters; some of the men whose penis was cut off were relatives of the participants. The size of a man’s penis was a factor that determined whether soldiers would cut off his penis. When perpetrators saw a man with a relatively large penis, they cut off their penis because they associated the man’s penis size with their manliness; they cut the penis off to strip them of what makes them a man. A common practice after cutting off a man’s penis was to put it in the penis owner’s mouth. In other cases, the perpetrators gave their victim’s penis to other people to eat, including some of the study participants. Mata experienced this and said that:

*“After they raped that boy, they cut his penis into pieces because they said it was too big for someone like him who is not a real man, and they forced us to eat it. When we realised that it is bad for you to eat human beings like you, we tried to escape”.*

### Genital beating

Male survivors also spoke of men’s penis and scrotum being beaten. Perpetrators ordered their victims to expose their penis, and they beat the penis with objects such as a bamboo stick or they kicked the men on their scrotum. Moses, a rape survivor, who witnessed and experienced this said:

*“When we were arrested again, they asked one of the boys with us to come, and they stripped him naked. They got his penis out, and they started beating it. The only thing they did to me* [during the second capture] *was that they kicked me on my scrotum. They kicked my scrotum three times”.*

### Insertion of objects into the anus

Participants also reported instances where their captors stripped them naked and inserted objects such as beer bottles or gun barrels into their anus. For some survivors, the objects were inserted primarily to inflict pain, while for others, the objects were used in a sex-like manner. For example, the barrel of a gun was inserted into Benz’s anus as a form of sex prior to being raped. Benz became visibly agitated while describing his experience. When this occurred, Benz was reminded that he could withdraw from the interview if he did not want to proceed. However, he opted to continue with the interview and provided details of his experience. According to Benz:

“*They removed my clothes completely and took me to the bush, naked. Now that time, I was thinking these guys were going to kill me, or they were going to rape me. Instead, they took this part of the gun that looked like a needle and put it inside my anus. Up to now, I cannot sit. They used it in my anus like this* [describing with his fingers] *as if they were fucking me. Then, after they used me with the other thing of the gun, they also raped me”.*

### Switching of traditional gender roles

#### Taking men as wives

Some South Sudanese men were captured and made to perform roles culturally associated with the role wives perform, in a deliberate attempt to emasculate them. Examples of such roles included being readily available to satisfy their captors’ sexual urges and doing domestic chores like cooking and washing clothes. Luis found himself in this situation, and according to him:

*“After they killed my wife, they told me I would have to stay with them. So, from there I went with them, and they made me like their wife. Any work, I was the one doing it. Any work like cooking food, I had to do it. Also, at night they rape me like a wife, and in the morning, I used to work like a woman”.*

### Forced witnessing of sexual violence

#### Men witnessing the rape of the women in their lives

Regardless of whether men were raped, some men were forced to observe either soldiers or rebel fighters rape the women in their lives, including their wives, mothers, daughters, and sisters. This was often done to show the men that they could not protect the women in their lives. For example, Essien was held at gunpoint while fighters raped his wife in his own house:

“*The rebels came to our house and called my name to come out. When I came out, I met four people outside. Two were behind me, and two were hiding near the door. They told me to sit down, pointed the gun at me, and I surrendered. Two entered the house, and I heard the sound of my wife. When I wanted to enter, they shot the gun in the air to fear and not to go inside, but I said I would rather die than see people rape my wife. So, I forced myself inside and met those two guys were raping my wife… those guys did it because they wanted to show me that they are men more than me’.*

After this event, Essien ended his marriage as he could not stand the shame of watching on while his wife was raped. His anger was obvious during the interview when he spoke of his desire to kill the perpetrators, if he found them.

Luis, who reported that he had been gang-raped (see above), narrated how he was tied to a tree to watch his wife being gang-raped before she was killed:

*“They told my wife to remove her clothes. Yea! Those guys were nine people. They raped my wife in front of me, and I was tied to the tree, and they forced me to see when they were raping my wife”.*

Mary, a humanitarian aid worker, said she had received reports by male refugees that the women in their lives had been raped in their presence during the conflict:

*“Some men will come to report with much fear and shame that “my wife and even myself were raped together”. First, the mother is raped, the wife is raped, the sister and then they raped him. We encountered some cases of that kind*”.

### Forcing men to cheer or assist during rape

Participants also reported that men were forced to applaud or assist (by holding victims down) perpetrators when they were raping other people. For example, Smalling shared an experience where he was caught in a group of other men, and the rebels picked some of the men to be raped, while others were forced to clap for the rapists or risk being beaten.

*“They just brought us like this and made us sit somewhere. Then they made us clap and cheer them as these boys were doing the act of raping the men they selected. So we had to cheer seriously. If you don’t do it, you are beaten”.*

In some instances, the people being raped were female members of the men’s families.

## Discussion

Interviews with 26 CRSV survivors and six humanitarian aid workers found eight direct and two indirect forms of CRSV perpetrated against male South Sudanese refugees who were displaced since the onset of the 2013 conflict and were living in Rhino and Imvepi resettlement communities in Uganda. Male-directed CRSV occurred in three settings: South Sudan, during flight to the resettlement community and within the resettlement communities. The forms of male-directed CRSV reported in this study are similar to the forms that other scholars have described in literature [8, 10, 11, 24], with a few exceptions. Forms of male-directed CRSV identified in the literature, but not reported in this study, include enforced sexual intercourse with dead bodies and enforced masturbation. The difference suggests that these forms of CRSV were perpetrated in other conflict settings, but not related to the South Sudan conflict that started in 2013. Moreover, this study found additional forms of CRSV that South Sudanese men experienced that were not previously reported in literature. Firstly, some men were forced, in captivity, to perform traditionally reserved roles for women (including but not limited to sexually satisfying the perpetrators) to emasculate the victims. South Sudanese men were also forced to cheer and assist perpetrators while soldiers or rebel fighters raped their compatriots and female family members. These two acts either occurred exclusively in the 2013 South Sudan conflict, or other scholars did not consider them as acts of CRSV and therefore, did not report them.

Although Ellsberg and colleagues reported on CRSV perpetrated against women during the 2013 South Sudan conflict [25], it is noteworthy that this is the first study to report extensively on CRSV perpetrated against men during the conflict. Identifying all the forms of CRSV reported by the survivors provides the necessary information for future studies to fully understand the impact and prevalence of CRSV perpetrated against South Sudanese men.

Feminist scholars have enhanced our understanding of CRSV as a gender subordination issue that occurs because of (or to establish) a power relationship between the perceived powerful masculine group and the perceived less powerful feminine group [10, 26–30]. The concepts of masculinity and femininity are socially constructed and exist in a spectrum rather than as a dichotomy [30–33]. Men who do not conform to the existing *hegemony* at any time are considered to exhibit *subordination* masculinity. Expectations of a person showing hegemonic masculinity include being strong, insusceptible to attacks, self-sufficient, and heterosexual [31, 34]. Sexual violence is a means of stripping men of their hegemonic masculinity [35]. Therefore, drawing on McDougall’s definition [36], any physical or psychological act that targets a man’s sexuality/sexual characteristics and aims to (or inadvertently) displace men from hegemonic masculinity should be considered sexual violence. The forms of CRSV described in this study satisfy these criteria because the perpetrators either told the participants that they were being violated to emasculate them or the survivors felt less than the socially construed masculine figure during and after their experience.

Moreover, the participants’ understanding and interpretation of the events and the observations noted by researchers during the interviews make it impossible to disregard their stories or discount their experiences as something else. For example, Smalling’s repeated involuntary movements to cover his genitals, despite being fully clothed, as he talked about being stripped naked, suggests that the event was psychologically traumatic for him. He described being forced to strip naked in the presence of other people as sexual torture. Similarly, although Essien and his wife were both raped (at different times), he noted that he felt more emasculated by being forced to witness his wife’s rape. The perpetrators intentionally forced him to watch the rape to displace him from the existing socially construed hegemony. His persistent anger and willingness to kill the perpetrators highlight why people like Essien must be supported; the act should not be trivialised by classifying it as something other than CRSV against Essien and his wife as survivors of indirect and direct CRSV, respectively.

There is no consensus in the literature on what constitutes CRSV. However, researchers, policy makers, post-conflict tribunals, international agencies, non-governmental organisations (NGOs) and humanitarian aid actors must incorporate the direct and indirect forms reported in this study into the definition and scope of CRSV against men. Understanding the different forms of sexual violence perpetrated against men in conflict and post-conflict settings and recognising them as such, is important for achieving justice for survivors. Unfortunately, forms of sexual violence, aside from rape, are often not considered to be acts of CRSV in post-conflict tribunals, like the International Criminal Tribunal for Yugoslavia and the International Criminal Tribunal for Rwanda, which were set up to prosecute perpetrators of wartime crimes [37]. Instead, they are either overlooked or downplayed to mere bodily harm [38]. Consequently, perpetrators of such acts of CRSV either avoid punishment entirely or are not punished commensurately because they did not rape the survivors, thereby denying survivors the justice they deserve. Post-conflict tribunals must be made aware of all the forms of CRSV described in this study and other similar studies already mentioned above, and the perpetrators must be punished accordingly. Access to appropriate justice can be therapeutic, as it reduces survivors’ tendency for negative emotions and would serve as a deterrent to potential offenders [39]. It is noteworthy that only the Special Court for Sierra Leone has prosecuted perpetrators of male rape in the case involving the Revolutionary United Front (RUF) [40].

Furthermore, healthcare practitioners and staff of NGOs that support refugees in humanitarian actions must be aware of the various forms of male-directed CRSV (beyond rape) in conflict and post-conflict settings to effectively support survivors. An awareness of their existence increases the likelihood that the practitioners will readily identify survivors of the different forms of sexual violence and institute appropriate care. For example, while a male refugee may not have been raped, he could have been stripped naked or forced to commit incest, which would negatively impact his health [41, 42]. When humanitarian aid workers focus exclusively on rape, they miss the opportunity to identify and then provide support to victims of enforced nudity and incest. Those victims will continue to suffer in silence or may attempt/decide to take their own life. Conversely, training healthcare practitioners and NGO staff to be proactive and ask male refugees about the experience of multiple forms of sexual violence, will help them promptly identify more (often overlooked) survivors.

### Limitations

The study was conducted with South Sudanese refugees in one district in Uganda, as authorized by the Office of the Prime Minister in Uganda. Therefore, to maximise this situation, the research team opted to conduct the study in a district that houses two large resettlement communities. However, it is possible that male South Sudanese refugees living in other districts or resettlement communities in Uganda experienced other forms of CRSV that our study participants did not report. Hence, conducting research on the same topic in multiple districts of Uganda would be valuable.

In addition, using the snowball sampling technique may have limited the forms of CRSV that participants reported because they may have nominated their friends and other CRVS survivors who had similar experiences. Despite this limitation, the use of the snowball sampling technique is appropriate in an exploratory qualitative study such as this, as survivors of CRSV are vulnerable and they are often difficult-to-reach populations [43]. Another sampling option would be a quantitative study with a randomly selected sample where questions are asked about more forms of sexual violence reported in this and other literature. However, it was important to first explore the subject, with this population, using a qualitative study.

## Conclusion

South Sudanese men who were displaced by the 2013 conflict reported ten different forms of CRSV (direct and indirect) that occurred either within South Sudan, during their flight to Uganda, or after arriving at the refugee resettlement communities. Previous studies have reported many of the forms of CRSV noted in this study. However, this study is unique because the primary participants are male survivors of CRSV that were not previously receiving support. Interviews with the male survivors of CRSV identified most forms of CRSV noted in the literature, plus additional forms of male directed CRSV which included, being forced to play roles traditionally ascribed to women and to cheer or assist during rape. Although some of the male survivors were not the direct victims of CRSV, the motivation for the acts, their relationships to the victims and the effects on the men, qualify them as indirect survivors of CRSV. Humanitarian aid workers, researchers and policy makers must be aware of all forms of CRSV to identify and support survivors of direct and indirect CRSV effectively. A discussion on the health consequences of CRSV against men is beyond the scope of this paper and will be addressed in a separate article.

## Data Availability

The datasets used and/or analysed during the current study are available from the corresponding author on reasonable request.
